# MEDIPS: genome-wide differential coverage analysis of sequencing data derived from DNA enrichment experiments

**DOI:** 10.1093/bioinformatics/btt650

**Published:** 2013-11-13

**Authors:** Matthias Lienhard, Christina Grimm, Markus Morkel, Ralf Herwig, Lukas Chavez

**Affiliations:** ^1^Departments of Vertebrate Genomics and Developmental Biology, Max Planck Institute for Molecular Genetics, Ihnestrasse 63-73, 14195 Berlin, Germany, ^2^Department of Rheumatology and Clinical Immunology, ^3^Institute of Pathology, Laboratory of Molecular Tumour Pathology, Charité Universitätsmedizin Berlin, 10117 Berlin, Germany and ^4^Division of Signaling and Gene Expression, La Jolla Institute for Allergy and Immunology, La Jolla, CA 92037, USA

## Abstract

**Motivation:** DNA enrichment followed by sequencing is a versatile tool in molecular biology, with a wide variety of applications including genome-wide analysis of epigenetic marks and mechanisms. A common requirement of these diverse applications is a comparison of read coverage between experimental conditions. The amount of samples generated for such comparisons ranges from few replicates to hundreds of samples per condition for epigenome-wide association studies. Consequently, there is an urgent need for software that allows for fast and simple processing and comparison of sequencing data derived from enriched DNA.

**Results:** Here, we present a major update of the R/Bioconductor package MEDIPS, which allows for an arbitrary number of replicates per group and integrates sophisticated statistical methods for the detection of differential coverage between experimental conditions. Our approach can be applied to a diversity of quantitative sequencing data. In addition, our update adds novel functionality to MEDIPS, including correlation analysis between samples, and takes advantage of Bioconductor’s annotation databases to facilitate annotation of specific genomic regions.

**Availability and implementation:** The latest version of MEDIPS is available as version 1.12.0 and part of Bioconductor 2.13. The package comes with a manual containing detailed description of its functionality and is available at http://www.bioconductor.org.

**Contact:** lienhard@molgen.mpg.de

**Supplementary information:** Supplementary data are available at *Bioinformatics* online.

## 1 INTRODUCTION

DNA enrichment methods are widely used for genome-wide identification of many different kinds of epigenetic marks. These techniques include chromatin-immunoprecipitation for localizing transcription factor binding sites or for revealing the genomic distribution of different histone modifications. Methylated DNA Immuno-Precipitation (MeDIP) (Weber *et al.*, 2005) and methyl-CpG binding domain (MBD) protein capture (Serre *et al.*, 2010) are similar techniques, but target the enrichment of DNA fragments containing methylated cytosines. Similarly, 5-hydroxymethylcytosines can be detected by antiserum specific to cytosine-5-hydroxymethylenesulfate (CMS) after treatment with sodium bisulfite (Pastor *et al.*, 2011). It can be expected that further affinity methods will be developed for immunoprecipitation (IP) of known or novel kinds of epigenetic marks. To provide a general framework for efficient genome-wide differential coverage analysis of IP-sequencing data, we have improved the user-friendly MEDIPS package. In contrast to the previous version, the MEDIPS update is capable of processing an arbitrary number of replicates or samples per condition. Furthermore, MEDIPS now integrates an elaborated statistical framework developed for the digital nature of count data, which includes a model for biological variation across replicates (Robinson *et al.*, 2010a), and has greatly reduced runtime and memory requirements.

## 2 MEDIPS WORK FLOW

**Fig. 1.**
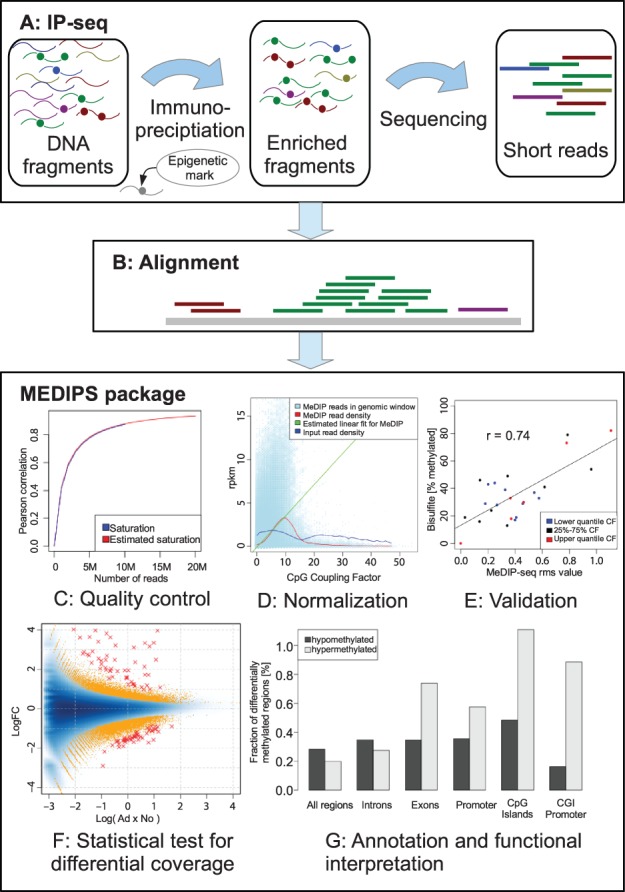
The MEDIPS work flow

The MEDIPS package provides functions for the quality control and analysis of data derived from IP-seq samples. It starts with the aligned reads (typically bam files) and can be used for any genome of interest. Figure 1 gives an overview of a typical work flow.

### 2.1 Preparation

In the first step, the alignment files (single- or paired-end) are imported, and the fragments overlapping previously specified genomic regions are counted. These regions can be either genome-wide windows of regular width or any given regions of interest. To control for polymerase chain reaction artifacts, MEDIPS optionally replaces reads with the same position and orientation by one representative.

### 2.2 Quality control

The saturation analysis helps to verify whether the given set of mapped reads is sufficient to generate a saturated and reproducible coverage profile of the reference genome. This is done by extrapolation of the correlation of subsets (see Fig. 1C).

To assess the effectiveness of the MeDIP/MBD enrichment, a function to calculate overall CpG enrichment is provided. MEDIPS identifies the fraction of CpGs in the reference genome covered by the sequencing data and evaluates their depth of coverage.

### 2.3 CpG density normalization

It has been reported by Down *et al.* (2008) that methylation levels obtained from MeDIP/MBD experiments and bisulfite sequencing cannot be compared directly. Therefore, MEDIPS maintains its normalization function based on the concept of CpG coupling analysis (Down *et al.*, 2008) to calculate the relative methylation score (see Fig. 1D and E). It has been shown by Chavez *et al.* (2010) that this normalization can improve the correlation to bisulfite data.

### 2.4 Differential coverage analysis

The main task for comparative epigenetic analyses is detection of regions with differential coverage between conditions. Variability, which can emerge from technical and biological variation, has to be estimated and modeled, and the statistical test has to consider the discrete nature of the count values. For this purpose, we make use of the edgeR package, which has been developed in the context of RNA-seq by Robinson *et al.* (2010a). It provides functions to estimate the biological variability from low number of replicates and models the count data using negative binomial distribution.

Alteration in copy number (CNA) are known to locally influence the MeDIP signal (Robinson *et al.*, 2010b). To control for this interference, alterations in copy number are evaluated and can be considered in further analysis.

To help with the functional interpretation of genomic regions identified by the differential coverage analysis, MEDIPS provides the functionality to annotate these regions with any provided set of annotations. The features can be imported from custom files, or from online databases, accessible from Bioconductor.

## 3 APPLICATION

To demonstrate the functionality of the MEDIPS package, we processed recently published MeDIP-seq data (Grimm *et al.*, 2013) that was generated to assess genome-wide epigenetic changes in mouse intestinal adenoma. For this study, differential methylation was inferred for the sample groups by calculating Wilcoxon rank tests for the normalized count values (reads per million, rpm) of each window. Differentially methylated regions (DMRs) were determined by applying filters for *P*-values, minimal coverage and ratios (Grimm *et al.*, 2013).

Here, we process the same data but by using the presented MEDIPS package version 1.12.0. The commented R script, showing the function calls of this analysis, can be found in the Supplementary Material. From the five adenoma and seven normal control mouse samples, 14–22 M MeDIP-seq reads were uniquely mapped to the mouse reference genome (NCBI37/mm9) using bowtie (Langmead *et al.*, 2009), of which ∼93% remain after replacing reads with the same position and orientation by one representative. The saturation analysis indicates sufficient sequencing depth, and the CpG coverage indicates an effective MeDIP enrichment (see Fig. 1C and Supplementary Figs S1 and S2). Comparison of the normalized relative methylation score values with bisulfite validation showed a good overall correlation of 0.69–0.79 with a set of bisulfite validation assays previously performed by Grimm *et al.* (2013) on the same genomic samples (see Fig. 1E and Supplementary Fig. S3). The edgeR test for differentially methylated regions finds 51.722 DMRs (*P* < 0.01), which correspond to 0.5% of the genome. Correction for multiple testing leads to 110 regions at 10% false discovery rate (FDR). Figure 1F shows the methylation logFC versus average log methylation (MA-plot). DMRs are depicted as orange points (*P* < 0.01) and red crosses (*FDR<*0.1). The result table containing the DMRs can be found in Supplementary Table S1. About 60% of the DMRs identified by Grimm *et al.* (2013) overlap with the DMRs identified by MEDIPS 1.12. A detailed comparison between the two approaches can be found in the Supplementary Material.

Although the overall number of hypo- and hypermethylated regions is balanced, preferential hypermethylation was found in functionally important subgenomic regions, such as promoters and CpG islands. In particular, CpG-rich promoters showed a substantial enrichment of hyper- over hypomethylation (5:1; see Fig. 1G). The identification of CpG-rich promoters as preferential targets for hypermethylation may provide important leads for further wet lab experiments. For instance, the analysis can be helpful to identify binding patterns of epigenetic modulator complexes and can be suited to identify candidate genes for epigenetic transcriptional silencing.

The processing of the aligned reads took ∼90 min on an AMD Opteron 6380 2.5 *GHz* computer, using 1 CPU core and allocating a maximum of 20 *GB* RAM.

*Funding*: German Federal Ministry of Education and Research with the grant EPITREAT (No. 0316190A) and by the Max Planck Society with its International Research School program (IMPRS-CBSC). Feodor Lynen postdoctoral Research Fellowship from the Alexander von Humboldt Foundation (to L.C.).

*Conflict of Interest*: none declared.

## Supplementary Material

Supplementary Data

## References

[btt650-B1] Chavez L (2010). Computational analysis of genome-wide DNA methylation during the differentiation of human embryonic stem cells along the endodermal lineage. Genome Res..

[btt650-B2] Down TA (2008). A Bayesian deconvolution strategy for immunoprecipitation-based DNA methylome analysis. Nat. Biotechnol..

[btt650-B3] Grimm C (2013). DNA-methylome analysis of mouse intestinal adenoma identifies a tumour-specific signature that is partly conserved in human colon cancer. PLoS Genet..

[btt650-B4] Langmead B (2009). Ultrafast and memory-efficient alignment of short DNA sequences to the human genome. Genome Biol..

[btt650-B5] Pastor WA (2011). Genome-wide mapping of 5-hydroxymethylcytosine in embryonic stem cells. Nature.

[btt650-B6] Robinson MD (2010a). edgeR: a Bioconductor package for differential expression analysis of digital gene expression data. Bioinformatics.

[btt650-B7] Robinson MD (2010b). Evaluation of affinity-based genome-wide DNA methylation data: effects of CpG density, amplification bias, and copy number variation. Genome Res..

[btt650-B8] Serre D (2010). MBD-isolated genome sequencing provides a high-throughput and comprehensive survey of DNA methylation in the human genome. Nucleic Acids Res..

[btt650-B9] Weber M (2005). Chromosome-wide and promoter-specific analyses identify sites of differential DNA methylation in normal and transformed human cells. Nat. Genet..

