# Supplementary material for: MEDIPS: genome-wide differential coverage analysis of sequencing data derived from DNA enrichment experiments
Source: Bioinformatics. 2013 Nov 13;30(2):284–6. doi: 10.1093/bioinformatics/btt650 (PMC3892689; doi:10.1093/bioinformatics/btt650)
Supplement: Supplementary Data [file supp_30_2_284__index.html]

MEDIPS: genome wide differential coverage analysis of sequencing data derived from DNA enrichment experiments — MEDIPS: genome-wide differential coverage analysis of sequencing data derived from DNA enrichment experiments — MEDIPS: genome-wide differential coverage analysis of sequencing data derived from DNA enrichment experiments — Supplementary Data 

# MEDIPS: genome-wide differential coverage analysis of sequencing data derived from DNA enrichment experiments

## Supplementary Data

files

**Files in this Data Supplement:**

- Supplementary Data - zip file
