# Supplementary material for: MEDIPS: genome-wide differential coverage analysis of sequencing data derived from DNA enrichment experiments
Source: Bioinformatics. 2013 Nov 13;30(2):284–6. doi: 10.1093/bioinformatics/btt650 (PMC3892689; doi:10.1093/bioinformatics/btt650)
Supplement: Supplementary Data [file supp_btt650_suppl_data.zip › comparison_dmr.pdf]

Comparing DMRs reported by Grimm *et al.* (2013) to the results of MEDIPS v 1.12.0

In the study by Grimm *et al.* (2013), the data was analyzed by counting sequencing read coverage (rpm values) for each sample at genome wide 500 bp windows that overlap by 250 bases. For each 500bp window, a p-value was calculated by applying the Wilcoxon rank test. Differentially methylated regions (DMRs) were determined by applying filters for p-values (< 0.01), minimal coverage ($rpm > 0.25$ in one group), and ratios ($3/4 > ratio > 4/3$). Using this approach, 17.690 DMRs have been detected (from now on refereed to as Grimm DMRs).

In contrast to this procedure, we now used MEDIPS 1.12 to count the reads at 250 bp windows, and applied a test based on the negative binomial distribution (Robinson *et al.*, 2010). As this test takes the distribution of count values into account, the difference of two sets of windows that are covered by only few reads is considered less significant than sets with higher covered windows with the same ratio. Hence, a threshold for the coverage is no longer necessary. Furthermore, the ratio is considered directly: The larger the difference between the groups, the more significant is the test. This is not the case for the non parametric Wilcoxon rank test. For this reason, there is no need for a ratio threshold.

Using only the p-value criteria, we found 51.722 DMRs with MEDIPS 1.12. However, in order to compare the DMRs from the two approaches, we additionally applied the logFC and coverage filter, which left 24.928 DMRs (from now on, these regions are refereed to as MEDIPS DMRs).

About 10.000 DMRs have been detected by both methods. This is about 60% of the Grimm DMRs, and 48% of the MEDIPS DMRs (Fig. 1).

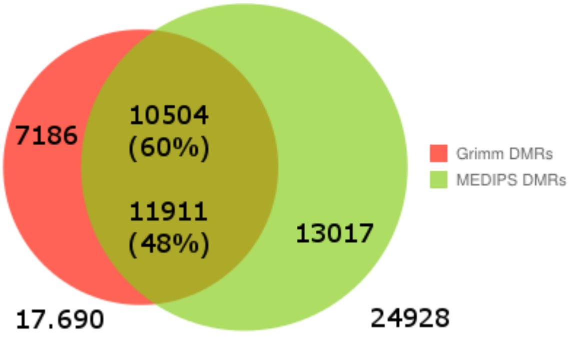

Figure 1: Overlap of DMRs found by the two approaches

The majority of the exclusive Grimm DMRs have a low read coverage. When stepwise increasing the coverage cutoff, the fraction of regions, also found by MEDIPS increases to 95% (Fig. 2). The fact that the test used in MEDIPS 1.12, that explicitly takes the distribution of the counts into

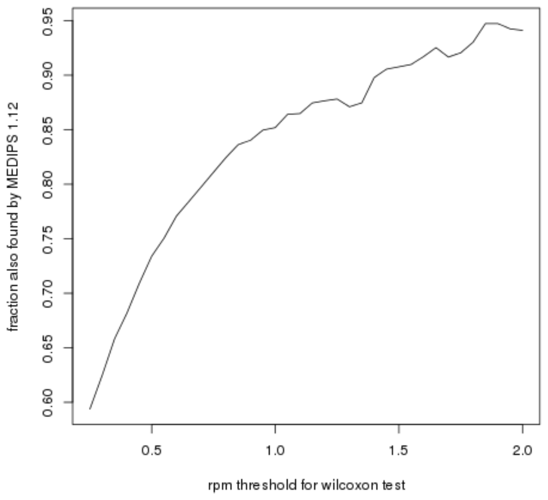

Figure 2: Dependency of overlap and read coverage.

account, is more conservative at low coverage, suggests that the ratio cut-off might have been too low, which led to false positive results.

About 52% of the MEDIPS DMRs have not been detected by the Wilcoxon rank test. We noticed that the Wilcoxon test is very susceptible to outliers. Figure 3 depicts a region where one of the adenoma samples has normal methylation, whereas all other adenoma samples show hypermethylation. The Wilcoxon test would give a p-value of 0.03 in this case, which is above the threshold for the study by Grimm *et al.* (2013). The ratio of the mean rpkm values of 7.8 : 1 has no influence on this p-value, since only the ranks are considered. In the new version of MEDIPS, this region would be considered differentially methylated ($p - value = 1.16e - 6$).

Further differences arise from the different window size: The smaller window size allows to find narrow peaks, that may be overseen using larger window size. On the other hand, if a larger window is moderately but consistently differentially methylated, it might be significant, whereas if it is split in two smaller windows, the test result might become negative.

Considering this comparison, we think, the statistical method used in MEDIPS 1.12 is advantageous over the Wilcoxon rank test, as the explicit modelling of count values leads to more statistical power. Furthermore, no rigid and arbitrary thresholds have to be applied.

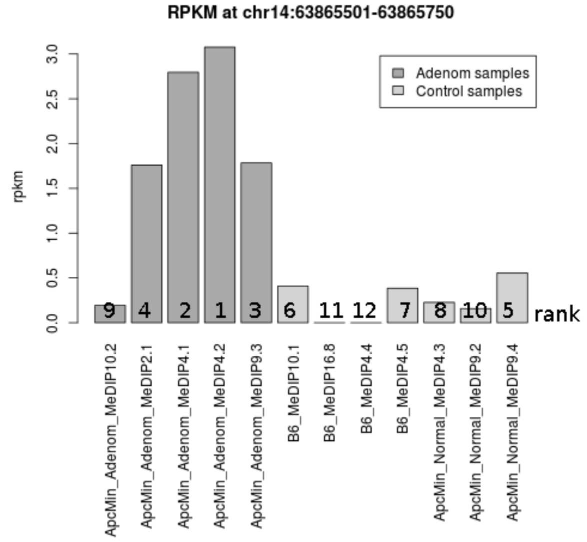

Figure 3: Example for MEDIPS 1.12 exclusive DMR

References

Grimm, C., Chavez, L., Vilardell, M., Farrall, A. L., Tierling, S., Bohm, J. W., Grote, P., Lienhard, M., Dietrich, J., Timmermann, B., Walter, J., Schweiger, M. R., Lehrach, H., Herwig, R., Herrmann, B. G., and Morkel, M. (2013). DNA-methylome analysis of mouse intestinal adenoma identifies a tumour-specific signature that is partly conserved in human colon cancer. *PLoS Genet.*, **9**(2), e1003250.

Robinson, M. D., McCarthy, D. J., and Smyth, G. K. (2010). edgeR: a Bioconductor package for differential expression analysis of digital gene expression data. *Bioinformatics*, **26**(1), 139–140.
