# Supplementary figures and images for: MEDIPS: genome-wide differential coverage analysis of sequencing data derived from DNA enrichment experiments
Source: Bioinformatics. 2013 Nov 13;30(2):284–6. doi: 10.1093/bioinformatics/btt650 (PMC3892689; doi:10.1093/bioinformatics/btt650)

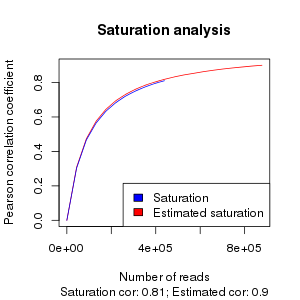

Supplement: Supplementary Data [file supp_btt650_suppl_data.zip › SupplFig1_saturation_B6_Min_ad_2.png]

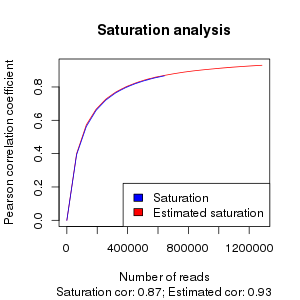

Supplement: Supplementary Data [file supp_btt650_suppl_data.zip › SupplFig1_saturation_B6_Min_ad_3.png]

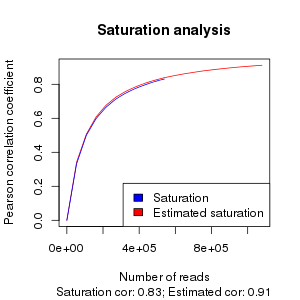

Supplement: Supplementary Data [file supp_btt650_suppl_data.zip › SupplFig1_saturation_B6_Min_ad_4.png]

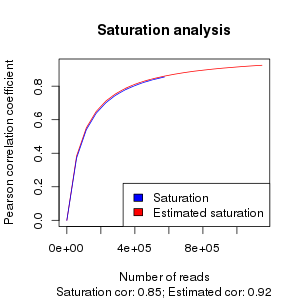

Supplement: Supplementary Data [file supp_btt650_suppl_data.zip › SupplFig1_saturation_B6_Min_ad_5.png]

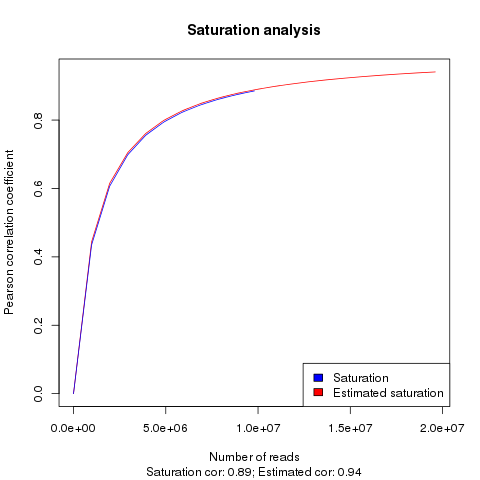

Supplement: Supplementary Data [file supp_btt650_suppl_data.zip › SupplFig1_saturation_B6_normal_1.png]

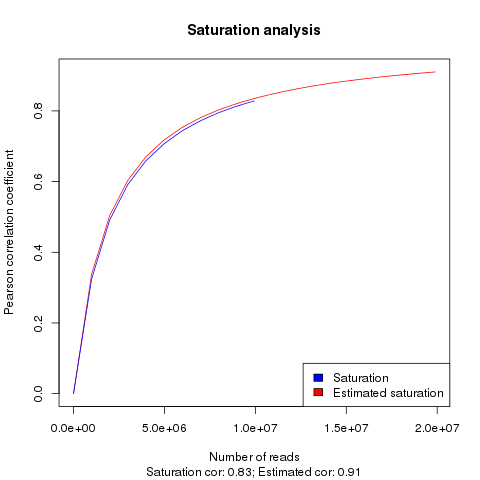

Supplement: Supplementary Data [file supp_btt650_suppl_data.zip › SupplFig1_saturation_B6_normal_2.png]

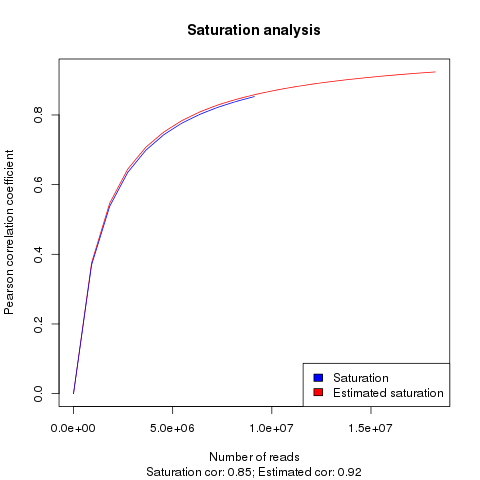

Supplement: Supplementary Data [file supp_btt650_suppl_data.zip › SupplFig1_saturation_B6_normal_3.png]

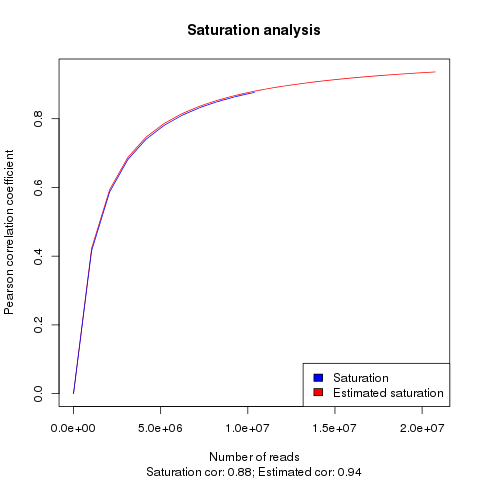

Supplement: Supplementary Data [file supp_btt650_suppl_data.zip › SupplFig1_saturation_B6_normal_4.png]

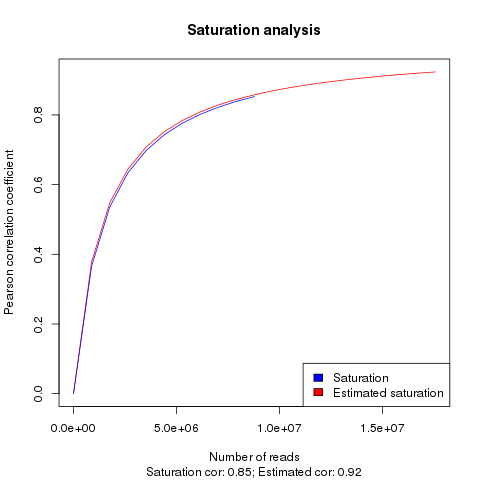

Supplement: Supplementary Data [file supp_btt650_suppl_data.zip › SupplFig1_saturation_B6_normal_5.png]

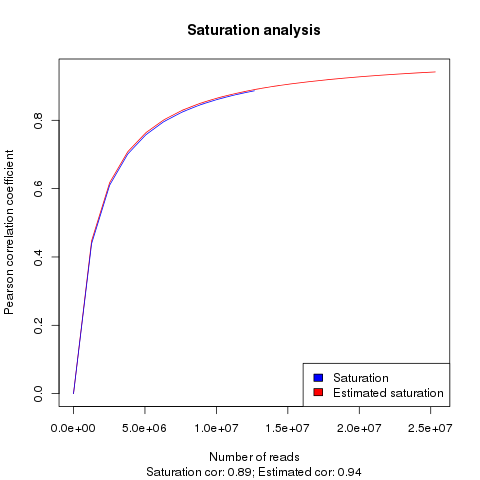

Supplement: Supplementary Data [file supp_btt650_suppl_data.zip › SupplFig1_saturation_B6_normal_6.png]

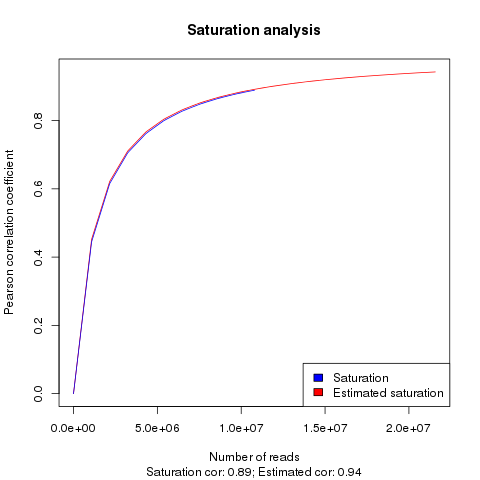

Supplement: Supplementary Data [file supp_btt650_suppl_data.zip › SupplFig1_saturation_B6_normal_7.png]

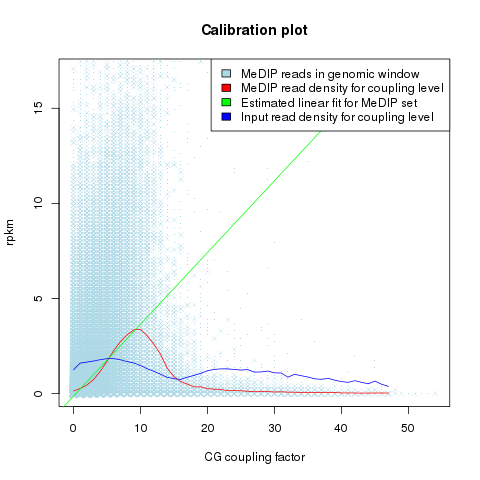

Supplement: Supplementary Data [file supp_btt650_suppl_data.zip › SupplFig2_calibration_B6_Min_ad_1.png]

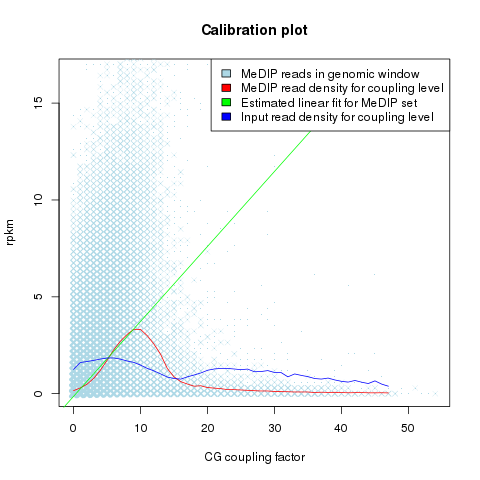

Supplement: Supplementary Data [file supp_btt650_suppl_data.zip › SupplFig2_calibration_B6_Min_ad_2.png]

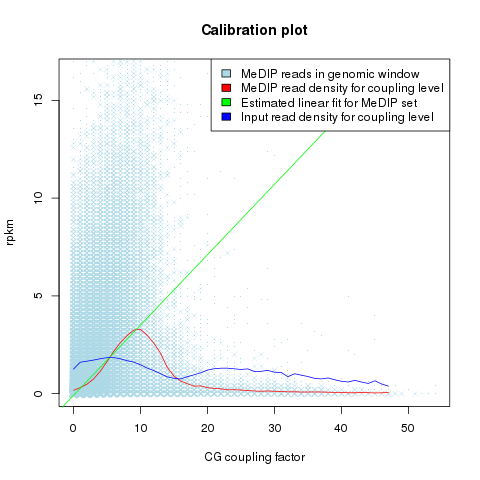

Supplement: Supplementary Data [file supp_btt650_suppl_data.zip › SupplFig2_calibration_B6_Min_ad_3.png]

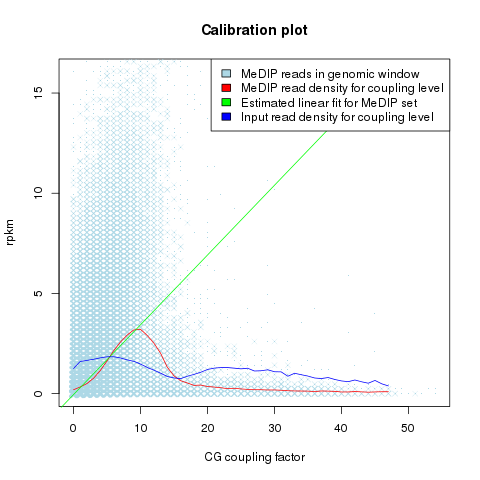

Supplement: Supplementary Data [file supp_btt650_suppl_data.zip › SupplFig2_calibration_B6_Min_ad_4.png]

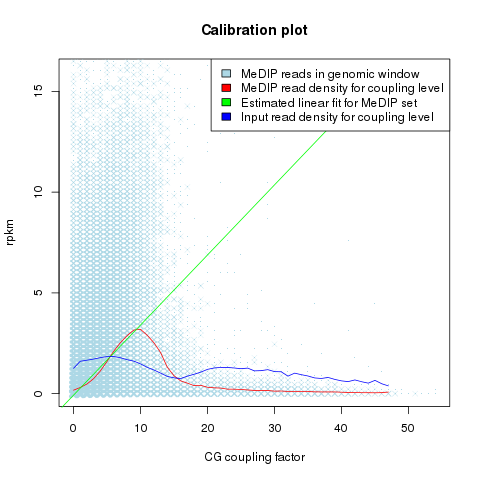

Supplement: Supplementary Data [file supp_btt650_suppl_data.zip › SupplFig2_calibration_B6_Min_ad_5.png]

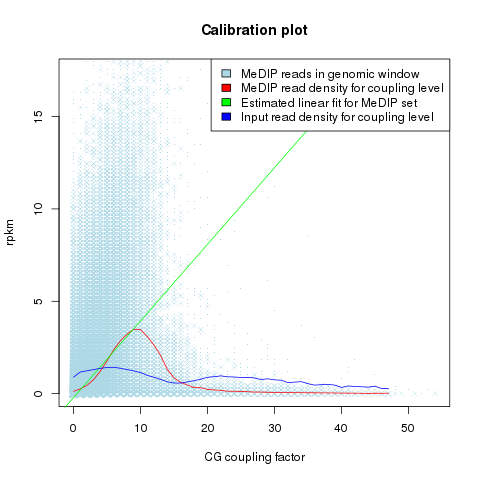

Supplement: Supplementary Data [file supp_btt650_suppl_data.zip › SupplFig2_calibration_B6_normal_1.png]

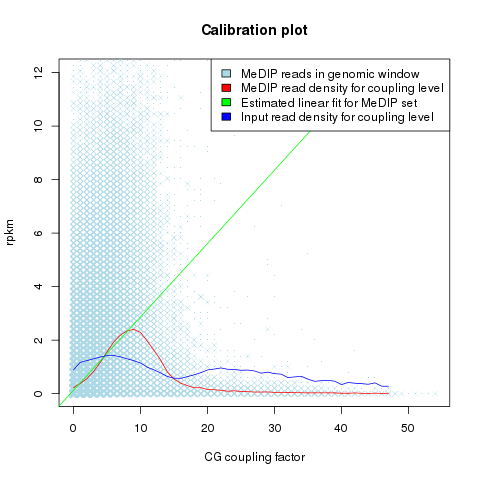

Supplement: Supplementary Data [file supp_btt650_suppl_data.zip › SupplFig2_calibration_B6_normal_2.png]

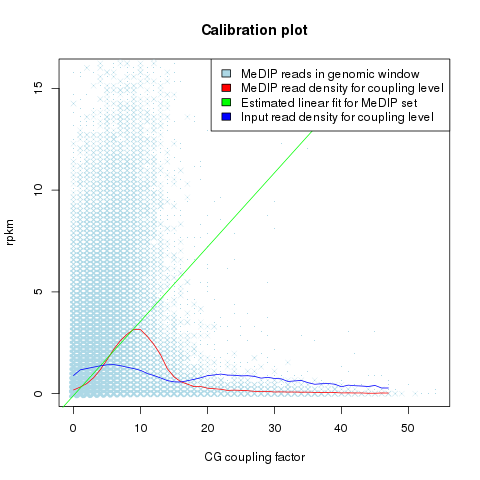

Supplement: Supplementary Data [file supp_btt650_suppl_data.zip › SupplFig2_calibration_B6_normal_3.png]

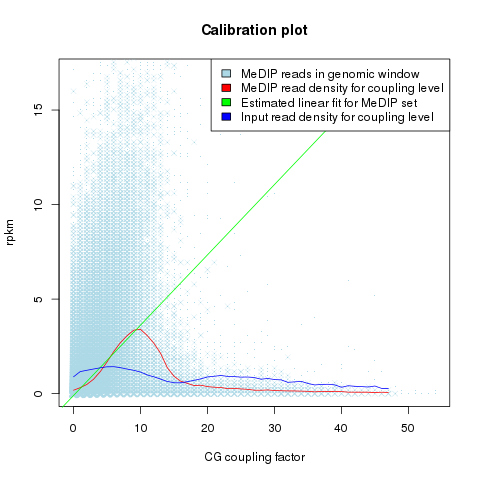

Supplement: Supplementary Data [file supp_btt650_suppl_data.zip › SupplFig2_calibration_B6_normal_4.png]

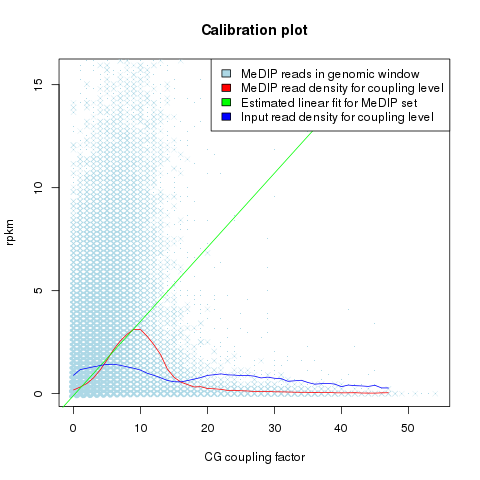

Supplement: Supplementary Data [file supp_btt650_suppl_data.zip › SupplFig2_calibration_B6_normal_5.png]

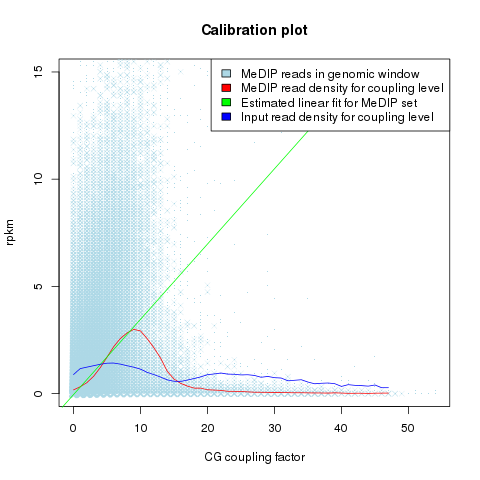

Supplement: Supplementary Data [file supp_btt650_suppl_data.zip › SupplFig2_calibration_B6_normal_6.png]

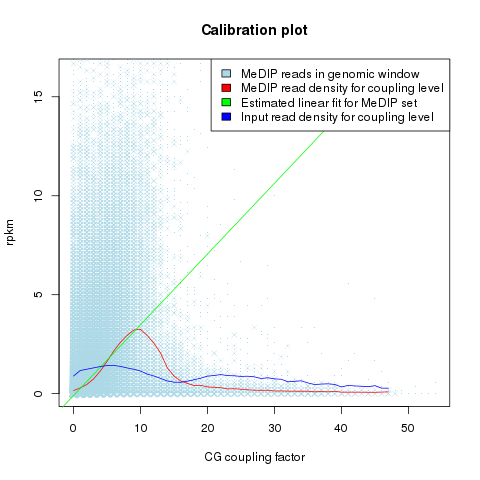

Supplement: Supplementary Data [file supp_btt650_suppl_data.zip › SupplFig2_calibration_B6_normal_7.png]

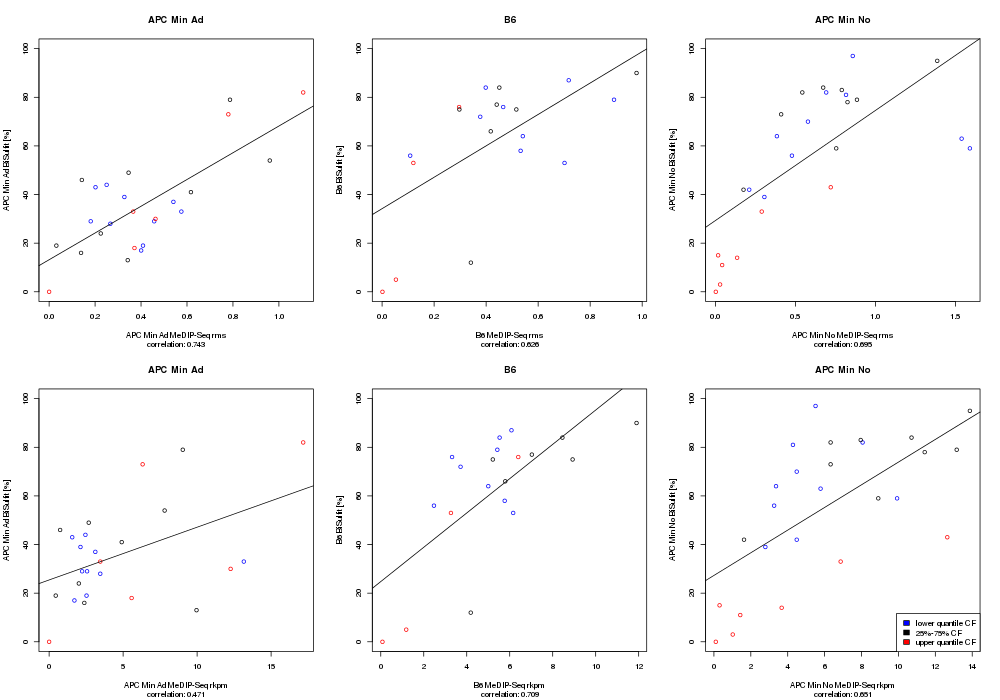

Supplement: Supplementary Data [file supp_btt650_suppl_data.zip › SupplFig3_mouse_BS_validation.png]
